# Grape by-products from the wine industry – an untapped sustainable resource for application in animal skin health preservation and treatment

**DOI:** 10.3389/fphar.2025.1620087

**Published:** 2025-08-26

**Authors:** Wanting Chen, Preeti Pandey, Zyta M. Ziora, Anjana Jayasree, Harendra S. Parekh

**Affiliations:** ^1^ School of Pharmacy and Pharmaceutical Sciences, The University of Queensland, Woolloongabba, QLD, Australia; ^2^ ARC Training Centre for Environmental and Agricultural Solutions to Antimicrobial Resistance (CEA-StAR), The University of Queensland, Brisbane, QLD, Australia; ^3^ Institute for Molecular Bioscience, The University of Queensland, Brisbane, QLD, Australia

**Keywords:** wine by-products, antimicrobial, animal skin infections, polyphenols, antimicrobial resistance, animal healthcare applications

## Abstract

The all-wine industry is projected to generate over US$528 billion in sales globally by 2025, and like many mass-producing industries, it too generates significant waste and by-products, much of which ends up in landfill. Among the various agricultural and industrial by-products, residues from winemaking stand out for their exceptionally rich and diverse bioactive compound content, primarily originating from grape skins, seeds and stems, all of which are rich in polyphenols, organic acids and tannins. These compounds have remarkable antioxidant, antimicrobial and anti-inflammatory properties and can therefore be diverted to agricultural, food preservation, cosmetic and pharmaceutical industries. The mechanism of action of the array of bioactive compounds includes disruption of microbial cell membranes, reduction of oxidative stress, and modulation of inflammatory responses. The current literature is limited to highlights of the scale of waste generated, and the application of its bioactive agents, however, it is notably absent of critical appraisal and discussion in sustainable avenues for development and value-added products, which are comprehensively elaborated herein.

## 1 Introduction

**FIGURE 1:**
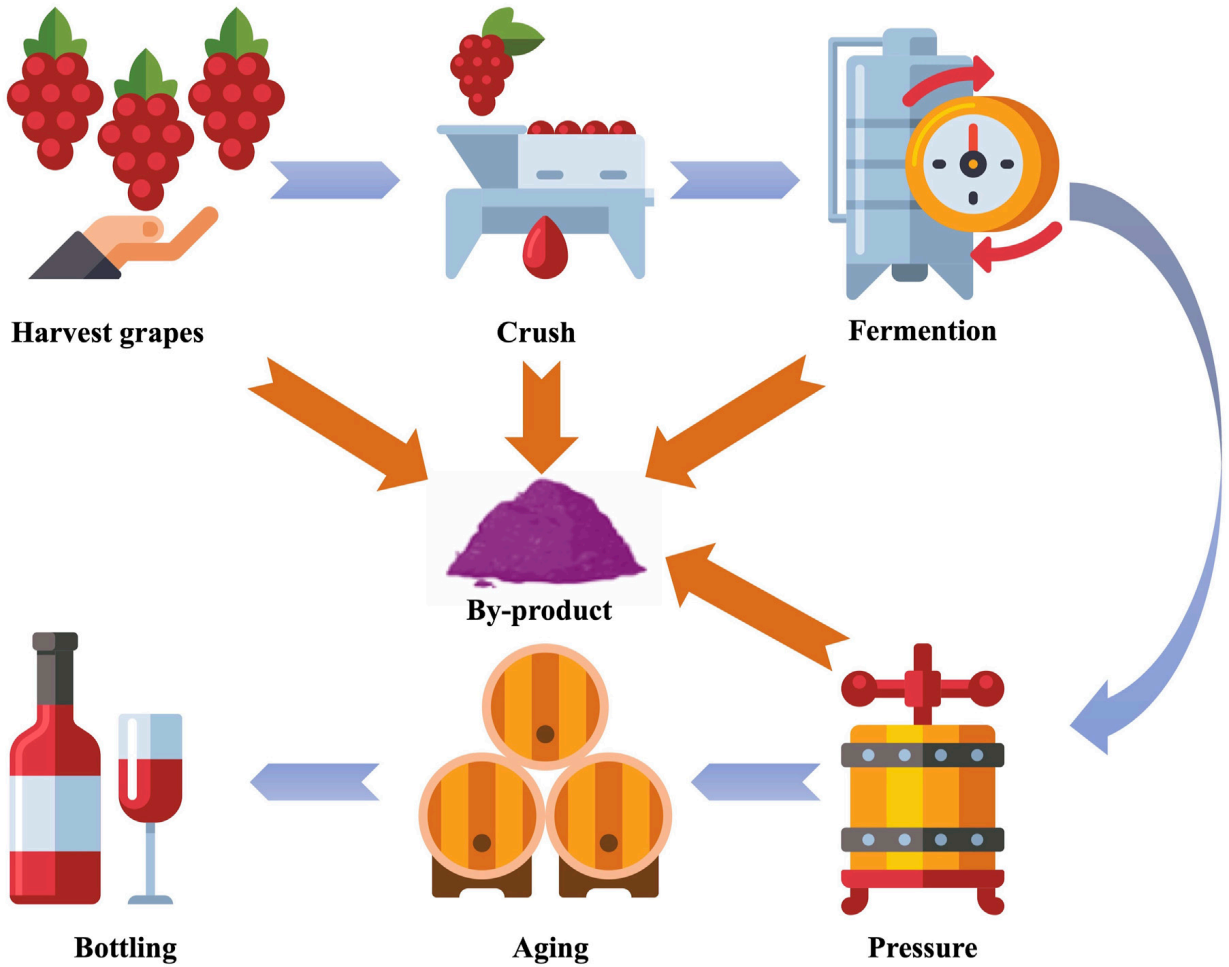
Schematic representation of wine production and by-product formation. Created with reshot.com.

Grapes (*Vitis vinifera*) are a widely cultivated fruit globally, especially of significant economic value in the winemaking industry. According to preliminary figures from the International Organisation of Vine and Wine global wine production in 2023 peaked at almost 24 billion litres (Wine, 2024). The waste produced during the winemaking process (Figure 1) represents about 30% of the total weight of the grapes used (Genisheva et al., 2023). By-products mainly consist of pomace (a mixture of skins, seeds and stalks), which, depending on the process, represents about 20% of the total weight of the grapes used (Angelini et al., 2022). These by-products were once considered as waste, but in recent years there has been a growing interest among scientists and industry in transforming wine by-products. By-products contain many bioactive compounds, especially polyphenols such as phenolic acids, flavonoids, and tannins. They have antimicrobial, antioxidant and anti-inflammatory properties, which have significant health benefits (Miklasińska-Majdanik et al., 2018; Corrêa et al., 2017). These by-products present potent candidates for application in agriculture, livestock production, and winemaking and are a potential source of plant-based antibacterial substances (Ferrer-Gallego and Silva, 2022; Nascimento et al., 2000).

Extensive research has been conducted on grape by-products to enhance human health. It is now becoming a promising solution in the veterinary field, showing potential in addressing the health and wellness challenges faced by the livestock industry (Baydar et al., 2004; Jayaprakasha et al., 2003).

The widespread prevalence of skin infections in domesticated animals and livestock is a cause for concern and the need for safe and effective treatments is on the rise (Joosten et al., 2020; Schrader et al., 2020). Animal skin infections are usually caused by bacterial pathogens such as *Staphylococcus aureus (S. aureus)*, *Streptococcus spp*., and *Pseudomonas aeruginosa (P.aeruginosa)*, causing serious health problems and significant economic losses to the livestock industries (Scharschmidt and Fischbach, 2013). Conventional treatment usually relies on antibiotics, but the effectiveness of antibiotics is gradually decreasing due to misuse and overuse, leading to antimicrobial resistance (AMR)-a global threat to human and animal health. For this reason, researchers are turning to natural plant alternatives to discover effective solutions that minimize the risk of AMR.

This review highlights the potential of grape by-products as an eco-friendly, sustainable, and naturally sourced alternative for controlling skin infections in animals. By summarizing existing knowledge on the bioactive compounds present in grape by-products, their antibacterial and anti-inflammatory mechanisms and formulation options for practical applications, this review aims to provide a comprehensive resource for future research and product development. Furthermore, it addresses the regulatory and standardization challenges associated with plant-based treatments, while assessing their applicability in large-scale animal healthcare. This exploration is intended to highlight the promise of grape by-products in not only improving animal health outcomes but also contributing to more sustainable livestock practices.

## 2 Structural basis and pathological implications of skin infections in domesticated animals and livestock

### 2.1 Structure and function of animal skin

**FIGURE 2:**
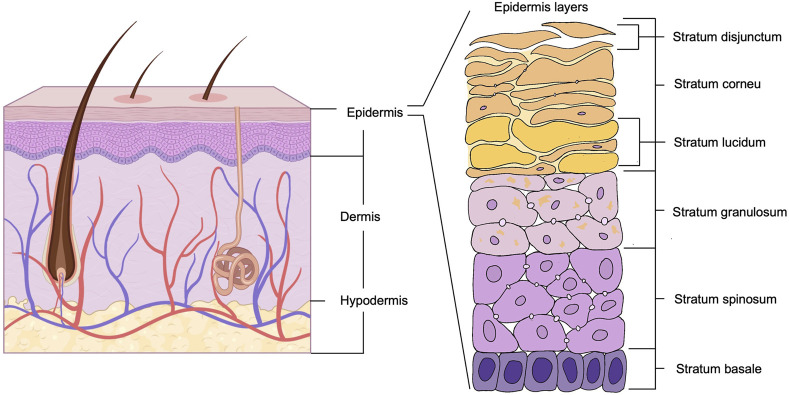
Schematic diagram of the multilayered structure of the skin. Created with BioRender.com.

The skin is the largest organ covering the entire body surface of humans and animals. It consists of three main layers: the epidermis, the dermis and the subcutaneous tissue (Souci and Denesvre, 2021). The structure of the skin is represented in Figure 2. The skin is the body’s first line of defence, protecting it from microorganisms, chemicals, and physical damage. The skin prevents the loss of water, electrolytes, and large molecules through the stratum corneum. As a barrier, it provides a stable internal environment for all other organs (Dehdashtian et al., 2018). The skin also plays crucial roles in sensation, temperature regulation, immune surveillance, secretion and excretion, and the production of vitamin D (Scott et al., 2003). Although the skin’s inherent functions enable it to withstand environmental stimuli, it remains susceptible to various forms of damage, compromising its structural and functional integrity. When the skin barrier is compromised, the risk of pollutants (pathogens) entering the body increases, heightening the likelihood of infection (Jonidi Shariatzadeh et al., 2025).

### 2.2 Microorganisms hosted on animal skin

Due to its structural characteristics, skin provides an environment conducive to the growth and reproduction of microorganisms. It plays host to millions of bacteria, fungi, viruses, and mites that form the skin’s microbiome (Roth et al., 1988), which plays an important role in protecting the host from pathogens, maintaining skin homeostasis, and promoting immune system development (Byrd et al., 2018; Christensen and Bruggemann, 2014). The microbial diversity on animal skin is much higher than that of humans, with the microbiome of parasitic species on an animal’s skin being influenced primarily by the host’s biological classification and geographic location. Different body parts also exhibit variations in microbial species (Ross et al., 2018). The skin can regulate bacterial composition, with some bacteria becoming dominant. For example, common bacteria found on farm animal skin include *Corynebacterium*, *Staphylococcus*, Aerococcaceae, *Psychrobacter*, *Micrococcus*, *Pseudomonas*, *Bacillus*, and *Acinetobacter* (Porcellato et al., 2020; O’Shaughnessy-Hunter et al., 2021). They constitute a natural microbiota that protects against pathogens. Companion animals’ skin is dominated by *Proteobacteria*, followed by *Actinobacteria, Firmicutes, Bacteroidetes,* and *Fusobacteria*, which also play important roles in skin health and immune responses (Older et al., 2017; Older et al., 2023). The majority of animal skin infections are caused by coagulase-positive *Staphylococci*, which are common bacterial pathogens responsible for various skin and soft tissue infections (Foster, 2012).

When the skin’s microbiome is disrupted by external factors (e.g., dysbiosis, reduced bacterial diversity), it negatively impacts skin health, leading to the development and progression of disease (Scharschmidt and Fischbach, 2013; Grice, 2015; Kong and Segre, 2012). In farm and companion animals, infections caused by skin damage are very common (Faccin et al., 2023) Table 1 lists common bacterial infections that lead to animal skin diseases.

**TABLE 1:** Common animal skin conditions of bacterial origin.

Skin region	Disease	Pathogens
Epidermis	Folliculitis	*Staphylococcus pseudintermedius (S. pseudintermedius*) *Staphylococcus sciuri* (*S. sciuri*) *Staphylococcus capitis* (*S. capitis*) *Staphylococcus chromogenes* (*S. chromogenes*) *Staphylococcus epidermidis* (*S. epidermidis*) *Staphylococcus aureus* (*S. aureus*) (Nagase et al., 2002)
	Superficial pustular dermatitis	*S. pseudintermedius* *S. aureus* *Escherichia coli,* (*E. coli*) *Pseudomonas* (Mauldin and Peters-Kennedy, 2016)
	Exudative epidermitis	*Staphylococcus hyicus* (*S. hyicus)* *Methicillin-resistant Staphylococcus aureus* (MRSA) *S. sciuri* *S. chromogenes* (Van Duijkeren et al., 2007)
Deep epidermis	Deep pyoderma	*S. pseudintermedius* *Staphylococcus schleiferi* (*S*. *schleiferi*) *Streptococcus* spp.*,* *Proteus* spp.*,* *Pseudomonas* spp.*,* *E. coli* *Bacillus* spp.*,* *Corynebacterium spp* *Pasteurella spp.* (Mauldin and Peters-Kennedy, 2016)
Dermis and Subcutaneous tissue	Mastitis	*Streptococcus agalactiae,* (*S. agalactiae*) *S. aureus* *E. coli* *Streptococcus pyogenes* *Klebsiella pneumoniae* (*K. pneumoniae*) *Pasteurella spp*. (Collado et al., 2016; Abdalhamed et al., 2018)
	Necrotizing fasciitis	*Streptococcus pyogenes* *Streptococcus canis* (*S*. *canis*) *S. pseudintermedius* *Clostridium perfringens* (*C. perfringens*) *Clostridium septicum* (*C. septicum*) *Clostridioides difficile* (*C. difficile*) (Sura et al., 2008)

### 2.3 Effects of bacterial infection on the epidermis

The epidermis is the outermost layer of the skin. According to its function and stratification, it is divided into five layers from the outside in: Stratum Corneum, Stratum Lucidum, Stratum Granulosum, Stratum Spinosum and Stratum Basale. Its primary cell type is the keratinocyte (Venus et al., 2010). The detailed structure of the epidermis is shown in the enlarged portion of Figure 2. The stratum corneum is composed of anucleated keratinocytes and lipid matrix (Menon et al., 2012), which has a high barrier function and is the first line of defence against water loss and invasion of external pathogens (Walters and Roberts, 2002). The spinous layer contains Langerhans cells, which are involved in the identification and removal of microorganisms and contribute to the local immune defence of the skin (Hibbs and Clark, 1959). The basal layer contains continuously dividing keratinocytes and melanocytes (Scott et al., 2003), which are involved in skin renewal and ultraviolet (UV) light protection (Plonka et al., 2009; Tapia et al., 2014).

When the epidermal barrier is compromised by trauma, moisture imbalance or immune deficiency, bacteria can easily colonize it and cause infection (Baquero et al., 2021). Common bacterial infections of the epidermis in animals include superficial pyoderma, pustules, and folliculitis, often caused by *S. pseudintermedius* or *S. aureus* in other species (Bloom, 2014). These infections usually present as pustules, papules, or epidermal ring of desquamation with clinical signs of itching, erythema, and desquamation (Loeffler and Lloyd, 2018). Although these infections may appear to be minor on the surface, they may develop into deeper dermal or subcutaneous tissue infections if not treated promptly. Chronic or recurrent epidermal infections are also often closely associated with underlying conditions such as atopic dermatitis, endocrine disorders or immunosuppression (Seckerdieck and Mueller, 2018).

### 2.4 Bacterial infections affecting the dermis

The dermis connects externally to the epidermis and internally to the subcutaneous tissue. It is rich in collagen and elastic fibers, which, together with connective tissue, provide the skin with elasticity and strength (Findlay et al., 2017). The dermis consists of two layers: the superficial papillary dermis and the deeper reticular dermis. It mainly consists of a collagen-based matrix containing a variety of cells, including fibroblasts, endothelial cells, and immune cells (such as macrophages, dendritic cells, lymphocytes, *etc.*) (Nguyen et al., 2009). The dermis encloses specialized structures like capillaries, nerve endings, sweat glands, and hair follicles, which nourish the skin through blood circulation. When bacterial pathogens breach the epidermal barrier, they invade the dermis, leading to more serious and potential for systemic infections (Chen et al., 2018). Common dermal bacterial infections in animals include abscesses and deep pyoderma, usually caused by anaerobic bacteria such as *Bacillus* spp., *S.* spp., and *Corynebacterium spp* (Lappin, 2012). These infections are typically characterized by pain, swelling, fever, ulceration, and localized bleeding (Loeffler and Lloyd, 2018). In some cases, systemic symptoms such as fever, loss of appetite, and lethargy may also occur, predisposing to sepsis (Mauldin and Peters-Kennedy, 2015).

### 2.5 Bacterial infections affecting subcutaneous tissue

The subcutaneous tissue, the deepest layer of the skin located beneath the dermis, is mainly composed of fat cells, collagen fibers, and loose connective tissue. It also contains blood vessels, sensory and motor nerves, and lymphatic vessels. The subcutaneous tissue plays a vital role in connecting the skin to underlying muscles and bones, as well as supporting important functions such as energy storage, insulation, and cushioning against external forces (Bichakjian et al., 2007). When this layer of tissue is disrupted by bite wounds, contamination from injections, surgical complications, or systemic immunosuppression, it becomes susceptible to bacterial invasion (Raskin, 2015). Subcutaneous infections in animals usually present as more serious conditions such as necrotising fasciitis, depending on the depth and spread of the infection. Common causative organisms include *S.* spp. and various anaerobic bacteria (e.g., *Clostridium spp*.) (Quilli et al., 2022). Unlike epidermal infections, subcutaneous infections usually require systemic antimicrobial therapy and surgical intervention such as drainage or debridement (Nolff and Meyer-Lindenberg, 2015). In addition, antimicrobial resistance of deep-tissue pathogens poses an increasing therapeutic challenge, thus raising concerns.

## 3 Conventional skin treatment methods

The use of antimicrobials and antibiotics is the main approach to treating animal skin infections. Gram-positive bacteria are responsible for most skin and soft tissue infections, especially coagulase-positive *Staphylococci*, with *S. aureus* and *S. pseudintermedius* being the most common pathogens (Faccin et al., 2023). Dependent on the infection site and severity, when topical antimicrobials cannot clear the infection, local antibiotic therapy or systemic treatment is used - Tables 2, 3 show the common treatment methods (Beco et al., 2013). Data on antibiotic dosage were obtained from Merck Veterinary Manual (Mercer, 2022).

**TABLE 2:** Topical antimicrobials commonly used to treat bacterial skin infections in animals, topical antibiotics and products.

Classification	Active Ingredients	Formulation	Available Products (Company, Country of Origin)
Topical therapeutic antimicrobials (Mueller et al., 2012)	Acetic acid, ethanol, boric acid, chlorhexidine, benzalkonium chloride, hydrogen peroxide, stannous fluoride, povidone-iodine, triclosan, silver sulfadiazine, phytosphingosine, ethyl lactate, benzoyl peroxide, zinc oxide	Antibacterial ointments and creams	Neogen Vetramil (Neogen Corporation, Lansing, Michigan, United States of America) Silver Honey™ Rapid Ear Care (W.F. Young, Inc., East Longmeadow, Massachusetts, United States of America) Zymox^®^ Topical Cream with Hydrocortisone (Pet King Brands, Inc., West Chicago, Illinois, United States of America)
		Antimicrobial spray	Vetericyn Plus^®^ Antimicrobial Wound and Skin Care Spray (Innovacyn, Inc., Oceanside, California, United States of America) Absorbine Silver Honey™ Hot Spot and Wound Care Spray (W.F. Young, Inc. East Longmeadow, Massachusetts, United States of America) Banixx^®^ Pet Care (Sherborne, Inc., Southern Pines, North Carolina, United States of America)
		Antibacterial lotions and wipes	Douxo^®^ Chlorhexidine PS Shampoo (Ceva Santé Animale, Libourne, France) Malaseb^®^ Medicated Shampoo (Dechra Pharmaceuticals, Northwich, Cheshire, United Kingdom) Pet MD Chlorhexidine Wipes (Pet MD, Illinois, United States of America)
		Gel	Pet Silver Antimicrobial Gel (Pet Silver, Florida, United States of America) KetoHex™ Antiseptic Gel (VetOne, Meridian, Idaho, United States of America)
Topical treatment antibiotics (DRUCKER, 2012)	Mupirocin, fusidic acid, mycopeptide, erythromycin, gentamicin, tetracycline, clindamycin	Antibiotic ointments and creams	Animax^®^ Ointment (Dechra Pharmaceuticals, Northwich, Cheshire, United Kingdom) Mupirocin Ointment (Various Brands, Petah Tikva, Israel) Bactroban^®^ Cream/Ointment (GlaxoSmithKline, Brentford, London, United Kingdom)
		Antibiotic Spray	Vetrimycin^®^ Antimicrobial Wound and Skin Care Spray (Innovacyn, Oceanside, California, United States of America) Fucidin^®^ Veterinary Spray (LEO Pharma, Ballerup, Denmark) Terramycin^®^ Wound Spray (Zoetis, Parsippany, New Jersey, United States of America)
		Antibiotic powder	Neo-Predef^®^ Powder (Zoetis, Parsippany, New Jersey, United States of America) Scarlet Oil Antiseptic Powder (Durvet, Blue Springs, Missouri, United States of America)
		Antibiotic Gel	Benzoyl Peroxide Gel (Various Brands, Lure, France) Keto-Med™ Medicated Gel (VetOne, Meridian, Idaho, United States of America)

**TABLE 3:** Systemic antibiotics, their scope of use in animals along with dosage and administration routes.

Systemic antibiotics (Beco et al., 2013)	Range of application	Antibiotic class	Antibiotic	Animal Species	Dosage and Route of Administration^a^
First-line antibiotics	Effective narrow- and broad-spectrum antibiotics that may have poor therapeutic efficacy (Van Vlaenderen et al., 2011)	Penicillin	Amoxicillin	Dogs, cats	11–30 mg/kg, PO, SC, or IV, every 8–24 h^b^
			Sodium penicillin G	Horses	10,000–20,000 U/kg, IV or IM, every 6 h
				Cattle	20,000 U/kg, IV or IM, every 6 h
			Ampicillin sodium	Cats	6.6–20 mg/kg, IV, IM, or SC, every 8–12 h
				Dogs	10–40 mg/kg, IV, IM, or SC, every 6–12 h
				Horses	15–40 mg/kg, IM or IV, every 6 h
				Cattle	4.4–11 mg/kg, IM, every 24 h, or 22 mg/kg, SC, every 12 h
		Cephalosporins (first generation)	Cefadroxil	Dogs, cats	22 mg/kg, PO, every 12 h
			Cephalexin	Cats	15–35 mg/kg, PO, every 6–12 h
				Dogs	15–45 mg/kg, PO, every 6–12 h
			Cefazolin	Dogs, cats	15–35 mg/kg, IM, SC, or IV, every 6–8 h
				Horses	10–20 mg/kg, IV, every 6–8 h; 15–20 mg/kg, IV, every 8–12 h (foals)
		Tetracyclines	Oxytetracycline	Dogs, cats	20 mg/kg, PO, every 8 h
				Horses	6.6 mg/kg, IV, every 12–24 h
				Cattle	6.6–11 mg/kg, SC, IM, or IV, every 24 h for up to 4 days 20 mg/kg, SC or IM, once
				Swine	6.6 mg/kg, IV, once 6.6–11 mg/kg, IM, every 24 h 20 mg/kg, IM, once
			Doxycycline	Dogs, cats	5–10 mg/kg, PO, every 24 h
				Horses	10 mg/kg, PO, every 12 h
		Sulfonamides	Sulfamethazine	Cattle, horses	225 mg/kg, PO, once, followed by 110 mg/kg, PO, every 24 h for 4 doses
				Calves, swine	247 mg/kg, PO, once, followed by 123.75 mg/kg, PO, every 24 h for 3 days as drench or drinking water
		Macrolides	Erythromycin	Cats	15 mg/kg, PO, every 8 h
				Foals	20–25 mg/kg, PO, every 6–8 h
			Tylosin	Cattle	17.6 mg/kg, IM, every 24 h for up to 5 days
				Swine	8.8 mg/kg, IM, every 12 h for up to 3 days
				Dogs, cats	6–20 mg/kg, PO, every 8–24 h
Second-line antibiotics	New broad-spectrum antibiotics to be used when first-line antibiotics prove ineffective	Cephalosporins (second and third generation)	Cefoxitin	Dogs, cats	20–30 mg/kg, IV, every 6–8 h
			Cefotaxime	Dogs, cats	50 mg/kg, IV, every 12 h
				Foals	20–40 mg/kg, IV, every 6–12 h
			Ceftiofur sodium	Horses	2.2–4.4 mg/kg, IV or IM, every 12–24 h; 10 mg/kg, IM or IV, every 6–12 h (foals)
				Cattle	1.1–2.2 mg/kg, IM or SC, every 24 h for 3–5 days
		Fluoroquinolones	Enrofloxacin	Cattle	7.5–12.5 mg/kg, SC, once 2.5–5 mg/kg, SC, every 24 h for 3–5 days
				Swine	7.5 mg/kg, IM or SC (postauricular), once
				Horses	5–10 mg/kg, IV, every 24 h; 5–10 mg/kg, PO, every24 h
				Dogs	5–20 mg/kg, PO, IV, or SC, every 12–24 h
				Cats	2.5 mg/kg, PO, IV, or SC, every 12 h; or 5 mg/kg, PO, IV, or SC, every 24 h
		Aminoglycosides	Gentamicin	Swine	1.1–2.2 mg/kg, PO as medicated water, every 24 h for 3 days
				Horses	7.7–9.7 mg/kg, IV, every 24 h
				Dogs	10–14 mg/kg, IM, IV, or SC, every 24 h
				Cats	5–8 mg/kg, IV, IM, or SC, every 24 h
Third-line antibiotics	Primarily reserved for multi-drug-resistant bacteria	Glycopeptides	Vancomycin	Dogs, cats	5–10 mg/kg, IV, every 8–12 h
		Polymyxin	Polymyxin B	Horses	0.6 mg/kg, IV, every 8 h
		Rifamycins	Rifampin	Dogs, cats	5–10 mg/kg, PO, every 24 h
				Horses	5–10 mg/kg, PO, every 12–24 h

^a^Data on antibiotic dosage were obtained from MSD MANUAL Veterinary Manual.

^b^Abbreviations: PO, Per os (oral); SC, Subcutaneous; IV, Intravenous; IM, Intramuscular.

Currently, improving the prevention of animal disease, treating infections, and promoting growth to target weights, heavily rely on the use of antibiotics (Woolhouse et al., 2015). Most of the world’s antibiotics are used in animals, not humans. In fact, as of 2017, about 73% of all antibiotics sold globally were used in livestock and other food animals. This reflects the animal industry’s heavy reliance on antibiotics to maintain health and productivity levels. Global consumption of veterinary antibiotics is significant and continues to grow: in 2020, ≈99,000 tonnes of antibiotics were used in animal feed, and, based on current trends, this is expected to increase to more than 8% per year by 2030 (Mulchandani et al., 2023). However, due to their improper use of antibiotic treatments, bacterial pathogens with antibiotic resistance genes have inevitably evolved (Martínez, 2012). In addition to antibiotics used for treatment purposes, the use of antibiotics as antimicrobial growth has greatly contributed to the development of antibiotic resistance in animals (van den Bogaard and Stobberingh, 1999). The spread of resistant bacteria subsequently raises public health issues for humans and the environment, causing significant pressure on animal welfare and the economy. According to the World Organisation for Animal Health, livestock-resistant infections alone could result in an annual loss of up to $1.7 trillion in global gross domestic product by 2050, and up to $5.2 trillion when the transmission of drug-resistant pathogens from livestock to humans is taken into account (through food, direct contact or environmental transmission) (Health WOfA, 2024).

Importantly, the impact of AMR is not limited to food animals; this applies to companion animals (pets) also. Dogs, cats and other pets are treated with antibiotics for infection and pre/post-surgery, and drug-resistant bacteria have become a serious problem in the field of companion animal medicine (Pomba et al., 2016). Antibiotic-resistant infections in pets can lead to long-term illnesses that are both difficult and expensive to treat. In many cases, drug-resistant infections in dogs or cats require extended hospital stays, multiple visits to the veterinarian, and expensive alternative medications or therapies. In addition, pets can be hosts or vectors for drug-resistant bacteria - for example, methicillin-resistant *S. aureus* (MRSA) and other superbugs can be transmitted between pets and people in the home, further complicating public health efforts (Caneschi et al., 2023). Antimicrobial resistance therefore encompasses both livestock and companion animals, reinforcing the concept of “one health”, where the health of animals, humans and ecosystems are interconnected.

Quantifying antibiotic use, reducing its usage, and finding alternatives are currently the most promising methods to manage antibiotic resistance. Among them, plant-derived bioactives (e.g., polyphenols extracted from winemaking by-products) are gaining attention due to their natural origin, lower risk of resistance development and multifunctional properties.

However, most of these alternatives are still at an early stage of validation and only very few have been converted to veterinary topical formulations. This gap highlights the need for continued innovation and formulation research to develop targeted, animal-safe and environmentally-friendly skin treatment products.

## 4 Biodiversity of wine by-products

Wine by-products mainly include grape pomace (skins, seeds, pulp and a small amount of grape stems), which are rich and varied in composition and have a high utilization value (Table 4). These by-products contain a large number of polyphenolic compounds, dietary fibers (soluble fibers, insoluble fibers) lipids (mainly unsaturated fatty acids such as linoleic acid and oleic acid) and vegetable proteins. They also contain some organic acids (e.g., tartaric acid, malic acid) and small amounts of residual monosaccharides (glucose and fructose). Minerals such as potassium, calcium and magnesium and trace elements are also present in these by-products (Karastergiou et al., 2024; Nirmal et al., 2025; Jin et al., 2019; Silva et al., 2024).

**TABLE 4:** Main components of wine by-products and their typical contents.

Ingredient categories	Content (% w/w)	Grape Origin
Moisture	50–65	Skin and seed and pulp
Dietary fiber	20–35	Skin and seed
Protein	10–15	Skin and seed
Lipids	4–13	Seed
Ash	4–7	Skin and seed and pulp
Carbohydrates	1–12	Skin and pulp
Polyphenol	4–11	Skin and seed
Organic acid	2–5	Skin and seed

### 4.1 Polyphenols in winemaking by-products: composition, bioactivity, and potential applications in skin health

Winemaking is a significant agricultural and industrial activity worldwide, generating large amounts of by-product. The biodiversity in these by-products is influenced by the grape variety, cultivation methods, and the winemaking process. Due to nuances in winemaking techniques, by-products from red and white wine production contain distinct phenolic compounds (Oliveira and Duarte, 2016).

Red wine is typically made from dark-skinned grape varieties (such as Cabernet Sauvignon, Merlot, Pinot Noir), where the skins and juice ferment together. White wine is usually produced from lighter-coloured grape varieties (such as Chardonnay, Sauvignon Blanc), and the grape pomace from white wine production does not undergo maceration, resulting in much lower anthocyanin (which gives the red colour to grape skins) content compared to red wine (Mosele et al., 2023). Grape pomace contains abundant plant secondary metabolites, specifically polyphenols, which make up about 10% of the dry weight of grape pomace (Karastergiou et al., 2024), These phenolic compounds are predominantly found in the seed (60%–70%), followed by skin (30%–35%), and flesh (10%) (Milinčić et al., 2021; Cotea et al., 2018). Most polyphenols in grapes belong to the flavonoid class, and they also include phenolic acids and stilbenes (Hegedüs et al., 2022; Kersh et al., 2023; On et al., 2022). Table 5 below outlines the common polyphenolic agents and their bioactivity as found in grape pomace.

**TABLE 5:** Common polyphenol compound classes, their origin, chemistry and bioactivity.

Compound class	Common name	Grape Origin	Chemical structure (chemical formula)	Bioactivity	References
Phenolic acid	Gallic acid	Skin and seed	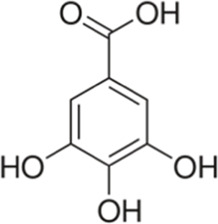 (C_7_H_6_O_5_)	Antioxidant, anti-inflammatory, anticancer digestive protection cardiovascular protection	Kahkeshani et al. (2019), Nayeem et al. (2016), Inoue et al. (2000)
	Caffeic acid	Skin and pulp	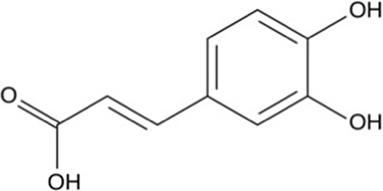 (C_9_H_8_O_4_)	Antibacterial, antioxidant, anti-inflammatory anticancer antiviral anti-diabetic immunostimulant cardiovascular protection liver protection	Espíndola et al. (2019), Pavlíková (2023)
	Vanillic acid	Skin	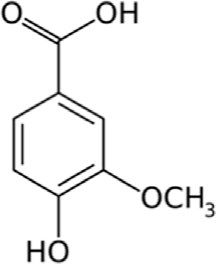 (C_8_H_8_O_4_)	Antibacterial, antioxidant, anti-inflammatory anticancer anti-diabetic anti-obesity cardiovascular protection	Kaur et al. (2022)
	Ferulic acid	Skin	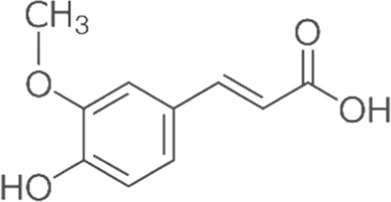 (C_10_H_10_O_4_)	Antibacterial, antioxidant, anti-inflammatory anticancer antiviral anti-allergic cardiovascular protection nerve protection liver protection	Kumar and Pruthi (2014), Alì et al. (2021), Mancuso and Santangelo (2014)
	Ellagic acid	Seed	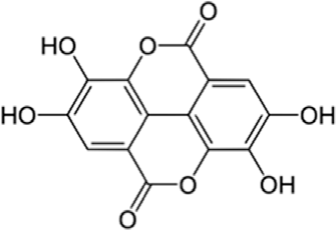 (C_14_H_6_O_8_)	Antioxidant, anti-inflammatory, anticancer cardiovascular protection nerve protection liver protection skin protection	Ríos et al. (2018), Sharifi-Rad et al. (2022)
	Syringic acid	Skin	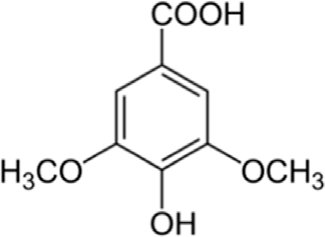 (C_9_H_10_O_5_)	Antioxidant, anti-inflammatory, anticancer anti-diabetic nerve protection	Srinivasulu et al. (2018)
Flavonoids	Quercetin	Skin	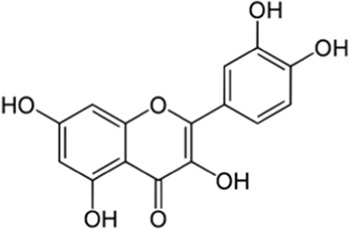 (C_15_H_10_O_7_)	Antibacterial, antioxidant, anti-inflammatory antiviral cardiovascular protection	Li et al. (2016)
	Catechin	Seed	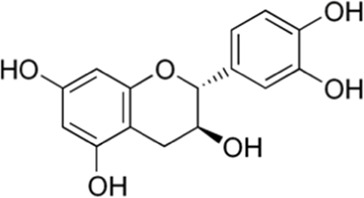 (C_15_H_14_O_6_)	Antibacterial, anti-inflammatory, anti-diabetic anticancer cardiovascular protection nerve protection liver protection	Baranwal et al. (2022), Shukla et al. (2018)
	Epicatechin	Seed	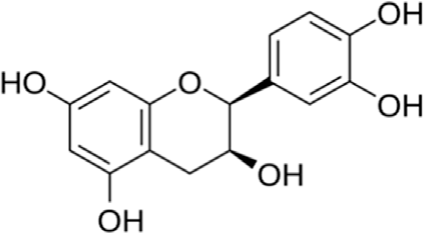 (C_15_H_14_O_6_)	Antibacterial, antioxidant, anti-inflammatory cardiovascular protection	Prakash et al. (2019)
	Rutin	Skin and seed	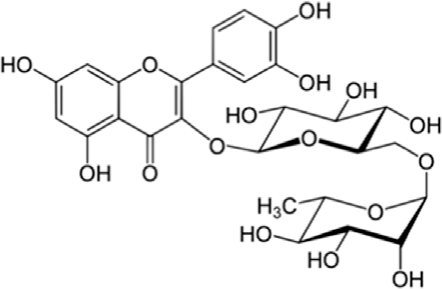 (C_27_H_30_O_16_)	Antioxidant, anticancer, cardiovascular protection nerve protection	Ganeshpurkar and Saluja (2017)
Stilbenes	Resveratrol	Skin	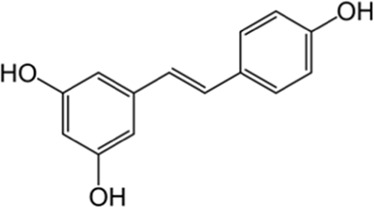 (C_14_H_12_O_3_)	Antioxidant, anti-inflammatory, anticancer anti-obesity cardiovascular protection nerve protection liver protection	Berman et al. (2017), Walle (2011)

Due to the different brewing processes of red and white wines, especially the pomace fermentation of red wines (Bocsan et al., 2022), the content of polyphenols in red wines is usually higher than that of white wines, resulting in significant differences in antioxidant activity and cardiovascular health (Fernández-Mar et al., 2012; Onache et al., 2023; Di Lorenzo et al., 2016). Polyphenolic compounds in red wine are effective in scavenging free radicals and protecting cells from damage caused by oxidative stress (Vejarano and Luján-Corro, 2022). Components such as resveratrol and quercetin can lower blood pressure, improve blood circulation and prevent atherosclerosis (Zhang et al., 2023). Polyphenols and tannins have an anti-inflammatory effect and help relieve chronic inflammation. Although white wines are lower in polyphenols, they still contain free radical reducing antioxidants (Bertelli et al., 2002). The acids found in white wines (such as tartaric and malic acids) help in the production of stomach acid, which promotes digestion. Its antimicrobial properties also help to maintain intestinal health (Tarko et al., 2020).

Numerous studies have characterized these compounds and underscored their strong antioxidant, anti-inflammatory, and antimicrobial properties. These bioactivities are highly relevant to skin health, where oxidative stress and microbial colonization are key contributors to the development of common conditions such as skin inflammation and wound infections. *In vitro* studies have demonstrated that polyphenols like pentagalloyl glucose and epigallocatechin gallate can effectively reduce oxidative stress and promote elastin deposition in the extracellular matrix, supporting skin structure and repair (Chowdhury et al., 2021). Polyphenols such as quercetin and resveratrol can rapidly exert antioxidant effects on the skin, mitigating oxidative stress at sites of inflammation by reducing reactive oxygen species activity within the biological barrier (Eskandari et al., 2019). One of the main uses of these polyphenols is the prevention and treatment of bacterial skin infections. Due to frequent exposure of the skin to a variety of pathogens, bacterial infections remain a significant and persistent health challenge. By understanding the structure of the skin and focusing on specific layers and regions that are susceptible to infection, the potential of polyphenol-based interventions for treating skin infections can be better assessed, utilising the unique bioactivities of winemaking by-products to support and maintain skin health.

## 5 Antimicrobial properties of grapes/wine by-products

Grapes contain a variety of secondary metabolites with antimicrobial properties, including phenolic acids, flavonoids and astragals, which impart a unique flavour to grapes and red wines. The antimicrobial components in grapes are mainly found in the pericarp, seeds and stems. They play a crucial function in plant defence against microbial attack and adaptation to environmental stresses. The diversity and complex structures of these compounds show a wide range of applications in the fields of food preservation, medicine and nutraceuticals. Thus, the antimicrobial activity of grapes and their by-products not only provides a direction for the reuse of their by-products but also offers a potential resource for the development of natural antimicrobial agents.

### 5.1 Major antimicrobial compounds in grapes and wine by-products

#### 5.1.1 Phenolic acid

Phenolic acids are polyphenolic compounds containing hydroxy acid groups, abundant in plants (including grapes and their by-products). They are renowned for their antioxidant, anti-inflammatory, and antimicrobial properties, benefiting both human health and industrial applications. In grapes, phenolic acids are primarily found in the skin, seeds, and stems, playing a significant role in the health benefits of grape products such as wine and grape extracts. Phenolic acids are mainly classified into two types: hydroxybenzoic acids (such as gallic acid, vanillic acid, and syringic acid) and hydroxycinnamic acids (such as caffeic acid, ferulic acid, p-coumaric acid, and sinapic acid). Phenolic acids are synthesized through the shikimate and phenylpropanoid pathways, which are essential for producing a wide range of plant secondary metabolites (Al et al., 2018; Kumar and Goel, 2019).

#### 5.1.2 Flavonoid

These are secondary metabolites widely present in various parts of plants, with most originating from the pigmented portions of plants. To date, thousands of flavonoid compounds have been identified in a wide range of sources, including fruits, vegetables, flowers, grains, seeds, nuts, herbs, spices, and plant-derived beverages such as tea, coffee, and wine (Daglia, 2012). Flavonoid compounds are characterized by a C15 carbon skeleton with a diphenylpropane structure. The structure includes two benzene rings (rings A and B) linked by a linear three-carbon chain. This central three-carbon chain forms a closed pyran ring (ring C) with the A ring, resulting in a chroman ring structure (the fusion of ring A with the pyran ring C) attached at positions 2, 3, or 4 to the second benzene ring (ring B). This structure defines the various types of flavonoid compounds (Rana and Gulliya, 2019; Kumar and Pandey, 2013). Flavonoids can be categorized into several subclasses—flavanols, flavones, flavanones, anthocyanins, flavanols, and isoflavones—based on the oxidation state of the central pyran ring. In nature, flavonoids protect plants from environmental stressors and serve as powerful antimicrobials or immune enhancers, helping plants defend against various pathogens (Górniak et al., 2019).

#### 5.1.3 Stilbenes

**FIGURE 3:**
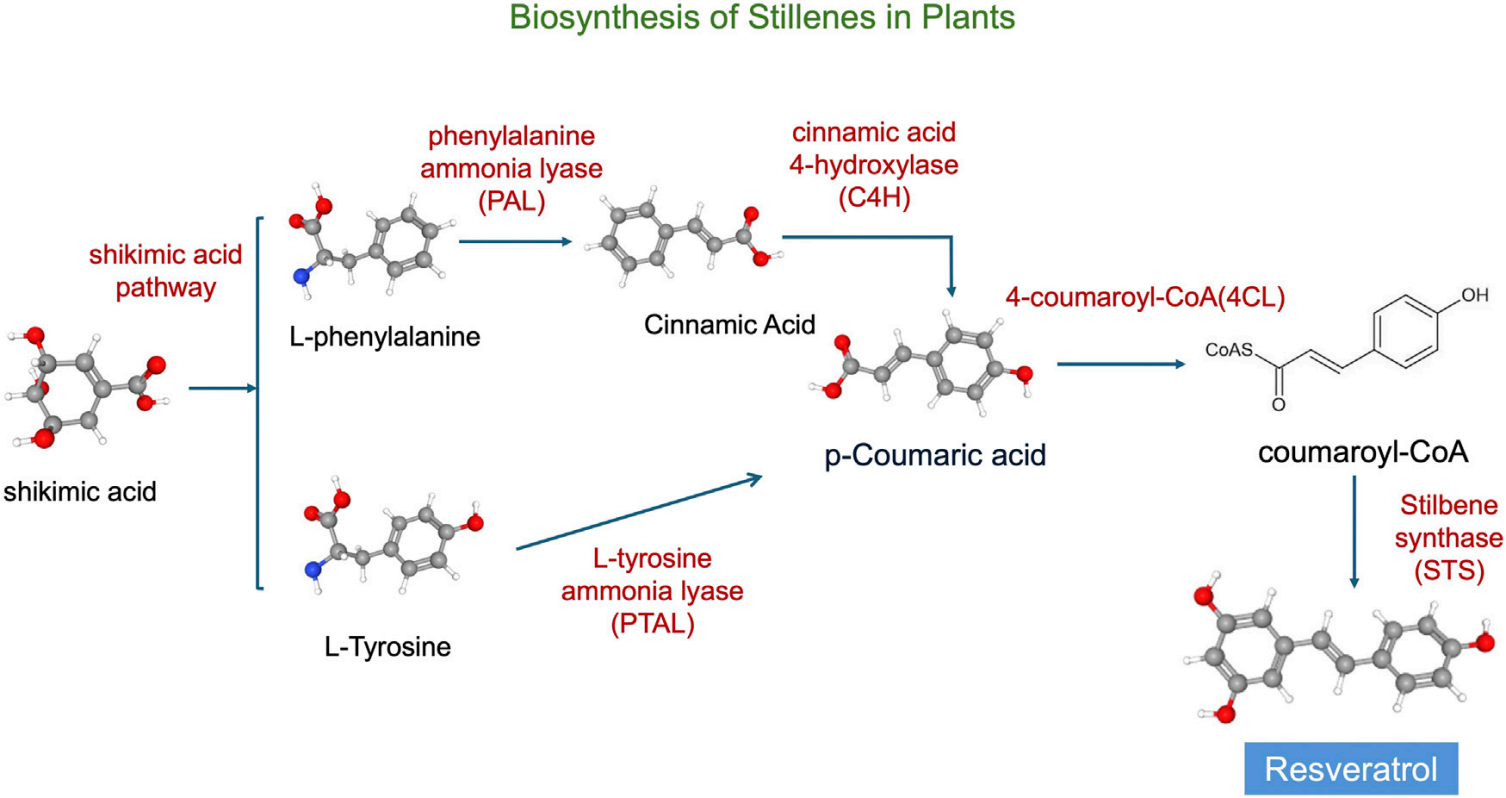
Synthesis pathways of stilbenes. The chemical structures in the figure are adapted from PubChem (National Center for Biotechnology Information, 2025).

These compounds are synthesized in plants via the phenylpropanoid pathway (Figure 3), beginning with the conversion of phenylalanine to cinnamic acid, which is then activated to cinnamoyl-CoA. Under the catalysis of stilbene synthase, cinnamoyl-CoA condenses with malonyl-CoA to form the stilbene backbone, such as resveratrol (Di et al., 2012; Kaur et al., 2024).

In addition, stilbene compounds effectively absorb or scatter UV radiation, reducing photo-oxidative damage (Niesen et al., 2013). Stilbene compounds undergo modifications such as glycosylation, methylation, prenylation and oxidative coupling to further enhance their antioxidant and antimicrobial activities. This synthetic pathway is stimulated under the influence of external factors (e.g., UV radiation, pathogen infection), which increases the accumulation of streptavidin compounds (Akinwumi et al., 2018). Stilbene compounds provide a variety of protective functions to plants under conditions of environmental stress (e.g., drought, low temperature, and UV radiation). They inhibit pathogens such as fungi and bacteria, helping plants to resist infections.

### 5.2 Mechanisms of antimicrobial action

**FIGURE 4:**
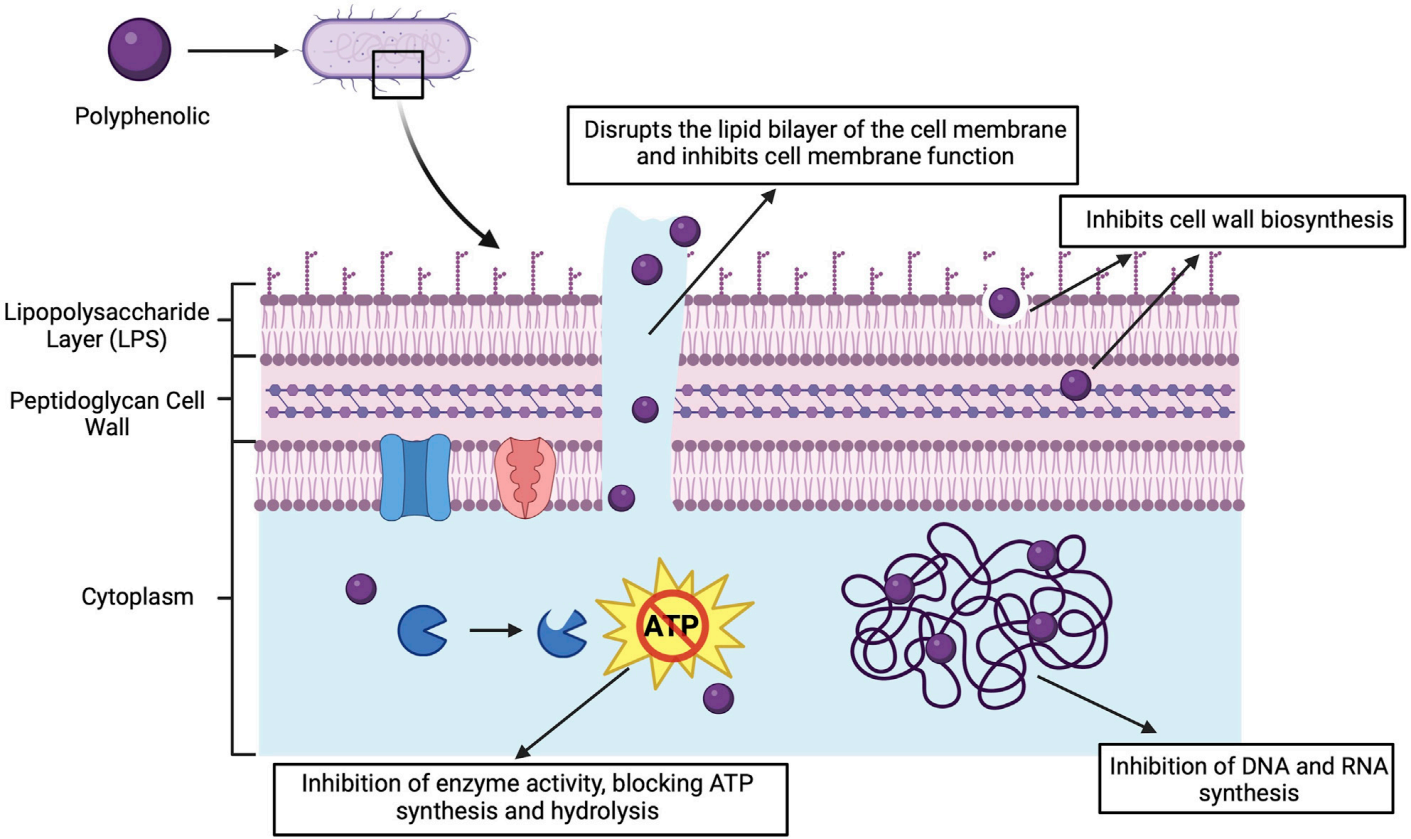
Antimicrobial mechanisms of polyphenolic compounds. Created with BioRender.com.

Polyphenolic compounds in grapes and their derivatives have a wide range of antimicrobial activities. Their antimicrobial mechanisms include disruption of cell membranes, inhibition of biofilms, interference with nucleic acid synthesis, alteration of membrane permeability, inhibition of toxins and restriction of movement (Figure 4). The following section describes these mechanisms in detail.

#### 5.2.1 Destruction of cell membranes

Polyphenolic compounds, represented by flavonoids, can directly insert into the lipid bilayer structure of bacterial cell membranes. Altered the stability of the membrane by insertion, making its structure loose and unstable and destroying the selective permeability of the membrane (Kumar et al., 2021). Leakage of cellular contents (e.g., ions, proteins, and nucleic acids) can occur as a result of damage to the cell membrane. This further affects the osmotic balance and energy metabolism of the bacteria, ultimately leading to bacterial inactivation or death.

Specifically, gallic and ferulic acids induce a non-polar character in *P.aeruginosa*. Under the influence of phenolic acid, electron acceptors on the surface of Gram-positive bacteria are increased, while the opposite effect is observed for Gram-negative bacteria. At concentrations up to 1,000 μg/mL, phenolic acids significantly enhance membrane damage and cellular content release in bacteria such as *E*.*coli*, *S. aureus*, *Listeria monocytogenes* (*L*.*monocytogenes)*, and *P.aeruginosa* (Borges et al., 2013). In addition, the fatty acid synthase type II (FAS-II) pathway is essential for cell membrane synthesis in Gram-negative bacteria. Various flavonoids such as quercetin, populin, baicalein, which can effectively inhibit FAS-II can prevent the synthesis of phospholipids and lipopolysaccharides by bacteria, thus thwarting cell membrane formation (Li and Tian, 2004).

#### 5.2.2 Inhibits the activity of a number of enzymes

Certain compounds in grapes inhibit toxins and degradative enzymes secreted by bacteria. By limiting the function of these toxins and enzymes, it is possible to reduce bacterial attack on host cells and prevent further development of the infection. Resveratrol reversibly binds to adenosine triphosphate (ATP) synthase and can inhibit ATP hydrolysis and synthesis processes in *E. coli*, thereby affecting its energy metabolism. This effect was particularly pronounced in the presence of non-fermentable carbon sources (e.g., succinate), which showed a significant limitation of bacterial growth, and in the presence of fermentable carbon sources (e.g., glucose), suggesting an inhibitory effect on the oxidative phosphorylation process (Dadi et al., 2009). Quercetin, catechin and EGCG inhibit bacterial DNA rotamase and block nucleic acid synthesis by binding to the ATP-binding site of the B subunit of the promoter of rotamase (Gradišar et al., 2007).

#### 5.2.3 Inhibition of biofilm formation

The active ingredients in grapes inhibit bacterial adhesion and biofilm maturation. Biofilms are highly organised communities of microorganisms that are widely found in nature. They can form protective structures on human surfaces or medical devices. This protective layer not only provides a stable internal environment for microorganisms to live in, but also greatly enhances their ability to adapt to external stresses (Kang et al., 2023). For example, biofilm formation enhances the resistance of microorganisms to antibiotics and disinfectants, while also enabling effective resistance to mechanical vibration and physical removal (Alonso et al., 2023). Antimicrobial ingredients limit the growth and spread of bacteria by preventing the formation of biofilms, destroying their structure or reducing their stability (Borges et al., 2016). Studies have shown that flavonoids in red wine inhibit the formation of *S. aureus* biofilms by binding to specific sites in bacterial cell membrane proteins through hydrogen bonding (Cho et al., 2015). Polyphenol extract from Rosa rugosa tea is effective in inhibiting *E. coli* and *P. aeruginosa* quorum sensing and biofilm formation (Zhang et al., 2014). Resveratrol can inhibit the biofilm of *E. coli* at concentrations 2–6 times lower than the minimum inhibitory concentration (MIC), and inhibit the biofilm formation of *S. aureus* at concentrations 3–4 times lower than the MIC (Lee et al., 2013). Resveratrol disrupts biofilm formation by affecting the expression of genes involved in quorum sensing, surface and secretory proteins, and extracellular polysaccharides essential for biofilm structure (Qin et al., 2014).

### 5.3 Factors influencing antimicrobial efficacy of wine by-products polyphenols

The amount and type of phenolic compounds in grapes (as the main raw material for wine production) and wine (as the final product), as well as in wine by-products (as production waste), are related to several factors. It is influenced by specificities including the variety of grape, growth and vinification, and ageing processes (Xia et al., 2010). Plant polyphenols demonstrate significant antimicrobial activity, and their efficacy varies depending on the type and concentration of polyphenol. The polyphenols recovered from dregs also vary depending on the technology, with dregs containing total phenols ranging from 1,200–4500 mg/L (Landeka et al., 2017; Dóka et al., 2023). Table 6 below lists the antibacterial power of some wine by-products against different bacteria.

**TABLE 6:** Polyphenol content and antimicrobial activity in wine by-products.

Grape Variety (Red/White grape)^a^	Origin	By-Product	TPC (mg GAE/g-)	Target Bacteria				References
				Gram-Positive	MIC (mg/mL)	Gram-Negative	MIC (mg/mL)	
Touriga nacional (R)	Portugal	Skins	35.5 ± 1.8	S. epidermidis	10	*K. pneumoniae*	100	Silva et al. (2018)
				B. cereus	100			
				*L. monocytogenes*	50			
				E. faecium	100			
		Stems	45.9 ± 10.7	S. epidermidis	75			
				*S. aureus*	100			
				E. faecium	100			
				*L. monocytogenes*	50			
		Seeds	261.3 ± 7.0	S. epidermidis	10	*K. pneumoniae*	50	
				*S. aureus*	50			
				*E. faecalis*	50			
				E. faecium	100			
				*L. monocytogenes*	10			
Preto martinho (R)		Skins	360.2 ± 2.5	S. epidermidis	10			
				*S. aureus*	75			
				*E. faecalis*	25			
				*L. monocytogenes*	50			
		Stems	363.0 ± 0.5	S. epidermidis	25	*K. pneumoniae*	75	
				*E. faecalis*	50			
				E. faecium	100			
				*L. monocytogenes*	50			
		Seeds	226.8 ± 6.9	S. epidermidis	25	*K. pneumoniae*	100	
				*S. aureus*	10			
				*E. faecalis*	10			
				*L. monocytogenes*	10			
				B. cereus	50			
Aglianico cultivar (R)	Italy	Pomace	57.1 ± 2.1	*S. aureus*	15	*E. coli*	40	Sateriale et al. (2024)
				*B. cereus*	20	*S. Typhimurium*	60	
Syrah var.(R)	Brazilian	Skins	759 ± 27	*S. aureus*	2.5	*E. coli*	1.25	de Andrade et al. (2021)
Pinot noir (R)	New zealand	Pomace	104.6 ± 18.8 to 291.1 ± 33.7	*S. aureus*	1.56–6.25	*E. coli*	12.5–25	Ye et al. (2022)
Riesling (W)			39.4 ± 1.2 to 239.4 ± 16.6	*S. aureus*	0.78–6.25	*E. coli*	3.13–25.0	
Cabernet sauvignon (R)	Chile	Pomace	12.577 ± 1.66 to 132.229 ± 4.8	*S. aureus*	No detailed value	*E. coli*	No detailed value	Sanhueza et al. (2014)
				*L. monocytogenes*				
Syrah (R)			6.989 ± 0.4 to 102.592 ± 3.7*	*S. aureus*		*E. coli*		
				*L. monocytogenes*				
Montepulciano (R)	Italy	Pomace	27.04 ± 0.13 to 199.31 ± 7.21	*S. aureus*	0.512–1.024	*E. coli*	1.024	Mollica et al. (2021)
				S. epidermidis	0.512–1.024			
		Seeds	12.03 ± 0.07	*S. aureus*	1.024	*E. coli*	1.024	
				S. epidermidis	1.024			
Tinto cão (R)	Portugal	Skin	No detailed value	*S. epidermidis*	100	*K. pneumoniae*	50	S et al. (2024)
		Seed		*L. monocytogenes*	50			
				*S. aureus*	75			
				*S. epidermidis*	75			
		Stem		*L. monocytogenes*	50	*P. aeruginosa*	50	
				*B. cereus*	25	*K. pneumoniae*	25	
				*S. aureus*	25			
				*S. epidermidis*	25			
		Shoot		*L. monocytogenes*	25	*P. aeruginosa*	25	
				*B. cereus*	50	*K. pneumoniae*	25	
				*S. aureus*	10			
				*S. epidermidis*	25			
		Leaf		*L. monocytogenes*	100	*K. pneumoniae*	75	
				*S. epidermidis*	50			

^a^Abbreviation: Red-R; White-W

In summarizing the aforementioned studies presented in Table 5, the climatic conditions of grape growing regions significantly affect the accumulation of flavonoids in their skin. The diurnal temperature difference affects the composition of compounds such as hydroxy anthocyanins and flavanols, while insufficient water increases the accumulation of anthocyanins (Zhu et al., 2017). Due to the paucity of studies on the antimicrobial activity of wine pomace and the multitude of research methods and research objectives, the correlation between the antimicrobial active compounds in wine pomace and their effects on bacteria has not yet been fully investigated. In particular, the types and content of antimicrobial compounds in wine pomace vary greatly depending on the extraction solvents, extraction procedures, part of the pomace contained, and grape variety have been found to affect the yield, polyphenol composition and antimicrobial activity of the extracts (Cheng et al., 2012; Oliveira et al., 2013). Therefore, it is difficult to draw uniform conclusions from the analysis of the available research data. The antimicrobial activity of phenolic compounds in grape pomace depends not only on the concentration but also on the specificity of the phenolic compounds (Xu et al., 2014).

Extracts isolated from common grape varieties (Table 6) showed strong inhibitory effect on Gram-positive bacteria (*S. aureus*, *S. epidermidis*, *B. cereus*). However, the inhibitory effect on Gram-negative bacteria such as *E*. *coli* was weak, and higher concentrations of the extracts were required to achieve an approximate effect. Gram-negative bacteria have a unique cellular structure and defence mechanism. Lipopolysaccharides make up the outer membrane of their cell wall, providing a sturdy shield against penetration of lipophilic solutions, a structure that Gram-positive bacteria lack. In addition, Gram-negative bacteria have an efflux pump system that expels the antimicrobial agent out of the cell, while secreting enzymes that degrade the antimicrobial agent, further diminishing the effect (Piddock, 2006). Gram-negative bacteria are also prone to forming protective biofilms that block antimicrobial and immune system attacks and rapidly spread resistance through gene transfer. These factors make Gram-negative bacteria highly resistant (Tavares et al., 2020).

Considering these findings, future research focuses on further developing the antimicrobial efficacy of phenolic compounds in grape pomace, especially against Gram-negative bacteria. In addition, investigating the synergistic effects of different phenolic compounds on antimicrobial activity, optimizing health-promoting compounds in wine by-products, advancing the use of grape pomace in natural antimicrobials, and supporting sustainable agricultural practices through the valorisation of winemaking waste is warranted.

## 6 Skin formulations of grape by-products in domesticated animals and livestock

### 6.1 Products currently used to treat skin ailments

In the scope of animal skin care products, most of the existing natural products are based on either plant extracts or oil components, such as aloe vera, coconut oil, tea tree oil and beeswax, to achieve gentle, symptomatic relief of skin problems. These products are typically applied for mild skin inflammation, dryness, and scratches, and have been validated to some extent against classical effects that promote healing (Table 7).

**TABLE 7:** Some commercially available products for domesticated and livestock animals. Animal skin health management.

Brand	Name	Ingredients	Formulation	Animal species	Indication	Dosage regimen	Regular price A$ (volume)
Natural Animal Solutions^®^	ItchyScratch Spray	Aloe Vera, Tea Tree Oil, and Golden Seal extract	Liquid spray solution	Dogs, cats	Provides immediate relief from pet itching and discomfort	1 to 4 times per day	$32.22 (100 mL)
	Dermal Cream	Comfrey oil Neem Seed oil Witch Hazel Golden Seal Yarrow Lavender oil Vitamin E Aluminium Acetate solution Grapefruit seed extract Rosemary extract	Cream	Dogs Cats, horses, and small animals	Improves skin appearance and moisturises	Once or twice daily	$31.55 (60 g)
	Dermal Oil	Comfrey oil Neem seed oil Lavender essential oil Eucalyptus oil	Oil	Dogs, cats, horses and other companion animals	Treats minor wounds and resists contaminants such as bacteria	Once or twice daily	$29.41 (100 mL)
BLACKMORES	PAW Manuka Wound Gel^™^	Leptospermum.sp (Manuka) honey (80%) and natural oils and waxes	Gel	Dogs, cats, horses and other companion animals	Sterile medical-grade wound dressing. Protects wounds, maintains their natural pH and reduces contamination and bacterial colonisation	depends on the clinical situation	$18.03 (25 g) $41.61 (100 g)
	PAW NutriDerm ^®^ Shampoo and Conditioner	Cerasine (ceramides and omega oils) Colloidal oatmeal	Shampoo, conditioner	Dogs, cats, horses and other companion animals	pH balancing and for dry and itchy skin	Use weekly	$25.27 (500 mL)
Shy Tiger	Soothe + Skin Spray Protect	Sage hydrosol; thyme hydrosol; Clove bud essential oil; Egyptian geranium essential oil; Kunzea essential oil; Lemon myrtle essential oil; Calendula essential oil; Copaiba essential oil; Cinnamon bark essential oil; Sweet Bay essential oil; Tocopherols – mixed (Vitamin E); Tea tree essential oil	Liquid spray solution	Dogs	Reduces inflammation, harmful bacteria and fungi. Disease relief for interdigital dermatitis, wound care and post-operative recovery	1–4 times daily	$30.90 (30 mL)
DR Show	GG Healer Essential Oil Cream	Tea Tree, Cedarwood, Chamomile, Rosemary, Patchouli, Ylang, Aloe Vera, Coconut and Pine Tar Oils	Cream	Horses	rubbed ears, girth galls and greasy heal Relieves itching and other skin problems, anti-inflammatory, antiseptic, soothes skin	Regular use	$49.45 (350 g)
	GG Healer Shake and Spray	Tea Tree, Cedarwood, Chamomile, Rosemary, Patchouli, Ylang, Coconut and Pine Tar Oils. Aloe Vera	Liquid spray solution	Horses	Anti-inflammatory, antiseptic, and soothing properties that may aid healing and recovery	Regular use	$42.95 (750 mL)
Plant Doctor	Neem Seed Oil	Neem Seed Oil	Oil	Horses, dogs, cats, cattle and other livestock	Soothes and heals inflammation caused by body rashes and other skin conditions	Regular use	$39.95 (1 L)
Stuart Products	Udder Green Rub	Arnica 1X, tea tree oil, menthol, corn mint and oregano oil	Lotion	Cattle	For use on inflamed and swollen nipples to reduce oedema (swelling) and to relieve cracked or injured nipples	After each milking	$50.96 (22 ounce)
Steuart’s	Udder Heal	Propolis tincture and comfrey extract	Ointment	Cattle	Promotes healing of cracked or injured nipples, wounds, papillitis lesions, nipple warts and breast rot	After each milking	$16.99 (8 ounce)
Dr. Paul’s Lab	LLC Super Wound Spray	Grain alcohol, aloe vera, garlic, comfrey, calendula	Liquid spray solution	Beef and dairy cattle, calves, sheep, goat and deer	Treatment and healing of minor cuts and abrasions	Repeat as needed	$35.18 (16 ounce)

### 6.2 Potential of grape extract in animal skin health maintenance

As the demand for natural ingredients for health applications increases, grape extracts have sparked much interest in the scientific community due to their diverse biological activities (Hegedüs et al., 2022). In recent years, a growing body of research has explored the positive effects of grape extracts on skin health with wound healing promoting effects demonstrated in several animal studies (Ajit et al., 2021). These active ingredients are able to effectively modulate the inflammatory response at the molecular level, inhibit the growth and reproduction of pathogens, and mitigate oxidative damage induced by wounds or UV irradiation through antioxidant mechanisms (Perde-Schrepler et al., 2013; Punzo et al., 2021). The application of grape extracts has shown potential not only in protecting the structural and functional integrity of the skin, but also in promoting tissue regeneration and accelerating wound healing. The studies in Table 8 systematically explores a wide range of possible applications of grape extracts in animal skin care, especially in different types of skin injuries and pathogenic infections.

**TABLE 8:** Grape and by-product extracts in animal skin health.

Source	Formulation	Animal species	Dosages	Applications	Results	References
Thompson seedless	Chitosan nanoparticles loaded with juice extract Ointment	Rats	Once daily	Wound healing Antifungal	The formulation has good resistance to fungi and has shown significant therapeutic effects on *A. niger* infected wounds in rats, with wounds healing completely within 7 days. The formulation is safe and effective, with no side effects such as inflammation or wound irritation	Elshaer et al. (2022)
Careh rooyieh	2% grape seed extract cream	Rabbit	Twice daily	Wound healing	2% grape seed extract cream promotes granulation tissue growth and epidermal reconstruction more effectively than clinical phenytoin. Wound healing was complete by day 13 with fewer inflammatory cells in the dermis	Hemmati et al. (2011)
Distrol^®^	Grape seed oil	Dog	Twice daily	Wound healing Antibacterial (multi-drug resistant bacterial) Anti-inflammatory	Use grapeseed oil to treat large, severe wounds (necrotic wounds with swelling) in dogs. Grapeseed oil was effective against multi-drug-resistant *Staphylococcus pseudintermedius* and *E. coli* in the wounds. The wounds contracted significantly within a week and closed completely in 28 days	Alice De Quadros et al. (2024)
Muscat of Alexandria grapes	Vaseline-based 2% grape seed extract ointment	Rats	Twice daily	Wound healing Antibacterial (*S. aureus* infection in diabetic rats) Anti-inflammatory	Grape extract Vaseline ointment was able to promote granulation tissue formation, accelerate wound healing in slower-healing diabetic rats, effectively combat *S. aureus* infection in wounds, and reduce oxidative stress. Ability to reduce levels of pro-inflammatory cytokines (e.g., TNF-α) and increase anti-inflammatory cytokines (e.g., IL-10 and TGF-β1), contributing to better immune response regulation	Hammam et al. (2022)
Cabernet sauvignon	Vaseline-based grape skin powder	Rats	Once daily	Wound healing	Enhanced wound contraction with grape skin powder treatment compared to treatment with mupirocin and control: complete wound contraction at day 13. Reduced epithelialisation time: the fastest epithelialisation time was observed in the grape skin treatment group, indicating the fastest skin regeneration. Higher levels of hydroxyproline in the treatment group, indicating increased collagen synthesis, a key factor in wound strength and structural support	Nayak et al. (2010)
Muscadine Grape	Grape Skin Extract, Grape Seed Extract, or a combination of both in 50% ethanolic extract	Rats	Once	Anti-inflammatory	These extracts significantly reduced inflammation, ear oedema and leukocyte infiltration in a mouse model of ear inflammation. The combined extracts had additional anti-inflammatory effects similar to those of the standard anti-inflammatory agent indomethacin	Bralley et al. (2007)
Burgund Mare	Grape Seed Extract in 40% acetone	Mice	Once daily	Against the photodamaging action Anti-inflammatory Antioxidant	Grape seed extract has a significant photochemical protective effect in the prevention of UV-B-induced skin damage. By modulating the activity of antioxidant enzymes (e.g., superoxide dismutase and catalase), decreasing the release of inflammatory mediators (e.g., IL-1β), and reducing DNA damage (e.g., the formation of cyclobutane-pyrimidine dimers), Grape seed extract was effective in reducing UV radiation-induced oxidative stress and inflammation. Furthermore, grape seed extract reduced the occurrence of apoptosis, suggesting its potential in protecting skin cells from UV damage and preventing photocarcinogenesis	Filip et al. (2011)

Although there have been numerous *in vivo* experiments demonstrating the effectiveness of grape extracts in wound healing, these studies have shown the potential of grape extracts as natural active ingredients in the field of animal skin health. However, most commercially available grape extract products focus on the field of human skin repair, with the main effects being antioxidant, anti-inflammatory and antibacterial, helping to slow down skin ageing, enhance skin barrier function, and improve skin radiance and elasticity (Sharafan et al., 2023; Ferreira and Santos, 2022). These products usually use grape seed or skin extract as the main ingredient and achieve their protective and repairing effects through the abundance of antioxidant actives such as polyphenols, proanthocyanidins and resveratrol (Soto et al., 2015). Despite the effectiveness of grape extracts in promoting skin health, similar products for animal skin treatment are virtually non-existent. This situation reflects the relative limitations in product development and ingredient selection in the current animal skin care field, especially in the utilization of grape extracts.

## 7 Challenges to use of grape extracts in animal skin treatment

### 7.1 Lack of in-depth studies on different species

Grape extract is a natural substance rich in bioactive compounds, with many studies having shown they are generally well-tolerated when applied topically to human skin (Fium et al., 2014; Benguechoua et al., 2025). In animal skin tests, resveratrol-containing hydrogels did not cause skin erythema and stratum corneum disruption in mice (Hung et al., 2008). Topical application of 2000 mg/kg of grape seed proanthocyanidin extract to the clipped intact skin of male and female albino rats for 24 h in a one-time dermal contact assay demonstrated that dermal application posed a very low risk of systemic toxicity at this dose (Ray et al., 2001). However, some animal studies have reported mild to moderate irritation in eye tests. Grape extracts, in particular, may induce a mild to moderate irritant response in the cornea or conjunctiva of rabbits, causing temporary discomfort or an inflammatory response (Bagchi et al., 2000). Such reactions are usually attributed to specific active ingredients in the extract, such as high concentrations of tannins or acidic polyphenols, and to the solvents used. Differences in irritation may be due to differences in skin barrier structure, absorption properties and sensitivity to phytochemicals.

The skin of different animal species varies greatly in structure, thickness and barrier properties. For example, the skin of dogs is thinner and more permeable than that of horses and is therefore more susceptible to both favourable and unfavourable effects of topical medications (Panakova et al., 2014). The development of effective products is complicated by the lack of standardised dosage regimens for different animals. Overdosing may lead to toxicity, while underdosing may render the product ineffective. Key factors such as frequency and duration of use can also affect efficacy and safety. Repeated use may cause irritation or sensitisation in animals with sensitive or damaged skin, and cumulative effects must be carefully assessed, particularly in animals prone to chronic skin conditions such as dermatitis.

### 7.2 Bioavailability and stability challenges

Grape by-products possess a wide range of bioactivities relevant to dermatological applications. Although these extracts are increasingly used in the food industry, their application as bioactive agents in topical formulations remains underexplored (Brown et al., 2006). Many polyphenols derived from wine by-products suffer from unfavourable physicochemical and pharmacokinetic properties, including low aqueous solubility, poor stability under improper storage conditions, and susceptibility to oxidation, all of which can lead to discoloration, odor changes, and diminished efficacy (Soto et al., 2015; Gonçalves et al., 2024). These compounds also tend to exhibit poor systemic bioavailability and limited tissue distribution.

Furthermore, grape extracts present notable formulation challenges. Their hydrophilic nature makes them poorly compatible with the lipid-rich environment of animal skin, impeding their ability to penetrate the stratum corneum and reach deeper dermal layers where therapeutic action is required (Spigno et al., 2007). Effective skin treatment depends on the bioavailability of active compounds—their ability to reach target sites at biologically relevant concentrations. However, the limited solubility and skin permeability of grape extracts, together with possible enzymatic degradation by the skin microbiota or topical metabolic processes, can significantly reduce their efficacy. In addition, the polyphenols in grape extracts are susceptible to oxidation and degradation when exposed to air, light and heat, thus affecting their stability and shelf life in formulations (Amendola et al., 2010). Animal grooming behaviours (e.g., licking or rubbing) and environmental exposures further challenge the stability and adhesion of topical products, and advanced formulations are required to meet these unique needs.

### 7.3 Insufficient standardisation of dosage and formulation

Inadequate standardisation of dosage and optimal formulation is a major challenge in the conversion of grape extracts into reliable and effective animal skin care products. Grape extracts contain a complex mixture of actives whose concentrations and proportions vary considerably depending on the extraction method, including solvent type, temperature and processing conditions (Table 9) (Barba et al., 2016). Dosage, formulation and frequency of use of extracts have not been standardised in existing studies. Therefore, it is challenging to develop dosages and formulations that are both effective and safe in animal care products. In addition, the bioavailability, skin absorption and stability of grape extracts need to be further optimised.

**TABLE 9:** Comparison of different extraction methods for grape extract components.

Extraction method	Polyphenols production	Chromoside production	Proanthocyanidin production	Flavonoids production
Solvent extraction	Moderate	Low	Moderate	Low
Ultrasound-assisted extraction (UAE)	High	High	High	High
Microwave-assisted extraction (MAE)	High	High	High	High
Supercritical fluid extraction (SFE)	High	Low	High	High
Pulsed electric field extraction (PEF)	Moderate to High	High	Moderate to High	High
Enzyme-assisted extraction (EAE)	High	High	Moderate	High

The production of high-quality grape extracts requires the use of specialised extraction and sealing processes to ensure the concentration and stability of the active ingredient. However, these processes typically increase costs, especially as the active ingredients need to be precisely separated and kept chemically unchanged during the extraction process. In addition, there are significant technical challenges to ensuring the consistency of extracts from different batches. For example, controlling variations in the quality of the raw material and the effect of changes in extraction conditions on the final product. These factors further limit the future need to optimise extraction and processing techniques in order to address these issues, as well as to develop more cost-effective methods to reduce production costs and improve the reliability of quality control.

### 7.4 Regulatory approval barriers

In many jurisdictions, animal care and treatment products must undergo a rigorous regulatory approval process, particularly when therapeutic claims are made. Grape extract-based formulations, due to their botanical origin and complex composition, are often classified as veterinary medicinal products when intended to prevent or treat disease. This classification necessitates compliance with strict regulatory frameworks. In particular, very few plant-based medicines are approved by the US Food and Drug Administration (US-FDA) for clinical use, because of their complex compositions, which makes it difficult to assess their safety, efficacy and bioavailability (Yo et al., 2022). As grape extracts are still emerging ingredients in animal care products, they require extensive experimental validation and regulatory approval to verify safety and efficacy. The time and economic costs associated with regulatory approvals limit their commercialization progress. To address these challenges and to ensure a more streamlined approach to evaluating veterinary drug products, the FDA provides a standardized set of guidance. FDA provides Target Animal Safety for Veterinary Pharmaceutical Products (FDA, 2009) is a standardized guideline for evaluating the safety of target animals It is referenced worldwide to assist in the development and registration of veterinary products. It aims to reduce the number of animal tests, lower the cost of research and promote the harmonization of global regulations. It also emphasizes the importance of research on scientific data, risk assessment to ensure the safety and efficacy of drugs in target animals. In the European Union, under the provisions of Directive 2001/82/EC (UNION TEPATCOTE, 2001), herbal veterinary products must also demonstrate safety and efficacy through clinical trials and only qualify for the simplified registration route under clearly defined conditions. In Australia, products with therapeutic effects are regulated by the Australian Pesticides and Veterinary Medicines Authority (Authority, 2015). If a grape extract formulation claims to treat skin infections or inflammation in animals, it must be registered as a veterinary medicinal product and undergo pre-market assessment. Despite the existence of these frameworks, there are currently no standardised international guidelines for topical grape extract preparations for veterinary use. The lack of harmonised standards has resulted in longer approval times and increased costs, thus hindering an appetite for product development.

### 7.5 Lack of marketing and consumer awareness of product effectiveness

The use of grape extracts in animal skin care products faces significant barriers due to limited marketing efforts and low awareness among consumer groups. Currently, most animal skin care products on the market still rely on widely known medicinal or botanical ingredients such as coconut oil, tea tree oil and aloe vera. These ingredients have earned the trust of a wide range of consumers and veterinarians because of the efficacy they have demonstrated over a long period of time during use. In contrast, grape extracts lack similar levels of awareness and trust as relatively new ingredients used in skin care, limiting their acceptance and broader use. A key factor contributing to this situation is the limited availability of established examples that can confirm the safety and efficacy of grape extracts in practical applications and the paucity of published data from animal clinical trials. This has led to a cautious approach to the use of new ingredients that do not have a reliable scientific basis. Although grape extracts have shown encouraging results in preliminary studies, their efficacy and reliability in animal skin care needs to be supported by more clinical trials and practical applications.

## 8 Future research directions

Future research should include comparative dermal pharmacokinetic and toxicity studies in representative animal species (e.g., dogs, cats, cattle, and horses) considering differences in skin anatomy and drug penetration in different species. This should be accompanied by the development of species-specific topical formulations and standardised dermal safety protocols. Additionally, the extraction process should be optimised to determine safe and effective dosages, eliminate irritating ingredients and support the development of formulations tailored to the specific needs of each species. Rigorous topical testing is necessary to refine these products and future studies should prioritise the assessment of skin tolerance to grape extracts in different animal species to lay the foundation for their use in veterinary dermatology.

To fully utilise the therapeutic efficacy of grape polyphenols in topical applications, the development of advanced delivery systems is essential. These can encompass phytosomes, sol-gel systems, liposomes, solid lipid nanoparticles (SLNs), nanostructured lipid carriers (NLCs) and polymer nanoparticles. Each of these systems offers unique benefits based on specific formulation goals. For example, phytomers enhance the lipophilicity of hydrophilic polyphenols and improve skin penetration through the formation of lipid-compatible complexes (Surini et al., 2018). Lipid-based carriers (e.g., SLN and NLC) provide containment, promote skin retention, protect antioxidants from degradation, and reduce systemic absorption (Santonocito, and Puglia, 2025). Sol-gel systems are FDA-approved and safe for human use, forming a bioadhesive matrix that extends skin contact for sustained and controlled drug release (Salem et al., 2025). At the same time, polymer nanoparticles allow for controlled release and targeted delivery, making them particularly attractive for the treatment of topical dermatological conditions (Salem et al., 2024). These delivery strategies not only improve the solubility and diffusion of polyphenols in the skin but also reduce systemic exposure and improve therapeutic indices.

To ensure consistent product quality, standardised extraction protocols, including solvent type, temperature and extraction time, and quality control markers for key bioactive compounds are required. The development of dosage guidelines specific to species, skin condition and disease severity can also help ensure safety and efficacy. Regulators can support this by promoting good manufacturing practice requirements for botanical veterinary products. Also harmonising international guidelines and creating simplified registration categories for botanical veterinary topicals can support rapid approval, across multiple markets.

To increase market acceptance, stakeholders should invest in publishing preclinical data, demonstrating real-world applications, and communicating the benefits of grape extract formulations to veterinarians and pet owners. Collaboration between academic researchers, veterinary professionals, and industry partners will help generate real-world success stories. Pilot projects or demonstration trials in veterinary clinics would serve to further enhance credibility and consumer confidence.

## 9 Conclusion

Grape by-products are a highly promising but under-utilised resource for the development of topical preparations aimed at improving animal skin health. These extracts are rich in polyphenols and other bioactive compounds with antioxidant, anti-inflammatory and antimicrobial properties that are highly relevant for dermatological applications. Going forward, more cross-species studies, rigorous safety assessments and optimised formulations are needed to validate the efficacy and safety of these natural compounds. There is also a need to harmonise regulation and increase market awareness to accelerate their widespread use. Through multidisciplinary collaboration and innovation, grape-derived topicals hold the promise of becoming effective and sustainable alternatives in veterinary dermatology.

In addition, the therapeutic effects exhibited by grape by-product polyphenols in animal models suggest that they have high translational value for human applications. Given the pharmacological properties of these natural compounds, their application in the treatment of human skin diseases such as atopic dermatitis, acne and photoaging could be further explored. Therefore, future research should also focus on bridging the gap between veterinary and human dermatology by investigating common skin pathophysiology and conducting comparative studies. Such cross-applications may pave the way for the development of novel plant-derived skincare interventions for both animals and humans, thus enhancing the relevance and impact of sustainable bioactive resources in modern medicine.

## Glossary

AMR: Antimicrobial Resistance
ATP: Adenosine Triphosphate
C. difficile: Clostridioides difficile
C. perfringens: *Clostridium perfringens*
C. septicum: *Clostridium* septicum
E. coli: *Escherichia coli*
EGCG: Epigallocatechin Gallate
FAS-II: Fatty Acid Synthase Type II
IM: Intramuscular
IV: Intravenous
K. pneumoniae: *Klebsiella pneumoniae*
L. monocytogenes: *Listeria* monocytogenes
MIC: Minimum Inhibitory Concentration
MRSA: methicillin-resistant *Staphylococcus aureus*
NLCs: Nanostructured lipid carriers
P. aeruginosa: *Pseudomonas aeruginosa*
PO: Per os (oOral)
S. agalactiae: *Streptococcus* agalactiae
S. aureus: *Staphylococcus aureus*
S. canis: *Streptococcus* canis
S. capitis: *Staphylococcus* capitis
S. chromogenes: *Staphylococcus* chromogenes
S. epidermidis: *Staphylococcus* epidermidis
S. hyicus: *Staphylococcus* hyicus
S. pseudintermedius: *Staphylococcus* pseudintermedius
S. schleiferi: *Staphylococcus* schleiferi
S. sciuri: *Staphylococcus* sciuri
SC: Subcutaneous
SLNs: Solid lipid nanoparticles
US-FDA: US Food and Drug Administration
UV: Ultraviolet
